# Enhancement of Ebola Preparedness across Africa

**DOI:** 10.3201/eid2212.160642

**Published:** 2016-12

**Authors:** Chloe E. Morozoff, David M. Pigott, Osman Sankoh, Sandra Laney, Simon I. Hay

**Affiliations:** Institute for Health Metrics and Evaluation, Seattle, Washington, USA (C.E. Morozoff, D.M. Pigott, S.I. Hay);; Wellcome Trust Centre for Human Genetics, Oxford, UK (D.M. Pigott, S.I. Hay);; INDEPTH Network, Accra, Ghana (O. Sankoh);; University of the Witwatersrand, Johannesburg, South Africa (O. Sankoh);; Njala University, Njala, Sierra Leone (O. Sankoh);; Vulcan, Inc., Seattle (S. Laney)

**Keywords:** Ebola virus, viruses, risk assessment, Ebola map, zoonotic disease, bats, transmission, one health approach, Ebola outbreak, zoonoses, Africa

Ebola virus disease (EVD) has demonstrated the devastating impact that zoonotic diseases can have on human populations (*1**,**2*). The recent EVD outbreak exposed national and international weaknesses in existing systems of outbreak response (*3*). The ability for this relatively rare virus to quickly spread merits the need for better preparedness plans in at-risk countries. Because zoonotic diseases account for most emerging infectious diseases (*4*), effective interventions must focus on animal reservoir hosts in addition to human health-seeking behavior and health system effectiveness.

A first step in preparing countries for a potential EVD index case is identifying areas environmentally suitable for zoonotic viral transmission. Previous research defined the spatial limits of the zoonotic niche of Ebola across Africa by considering several environmental co-variates and hypothesized reservoir bat species (*5**,**6*), and this niche map was recently updated in consideration of new data (*7*). The updated zoonotic niche map identified 23 at-risk countries having areas environmentally suitable for Ebola. Additional analyses permitted the mapping of population vulnerability and health system capacity to respond to a spillover event (Table 1).

**Table 1:** Description of spatial epidemiology of EVD tool layers*

Title	Function	Potential application	Availability
Predicted distribution of Ebola virus disease across Africa	Users can view the 23 countries with areas of environmental suitability for EVD as well as previous outbreak occurrence data points (*8*).	Countries with predicted environmental suitability can initiate or better target surveillance, emergency operations, and preparedness measures.	http://vizhub.healthdata.org/ebola/
Predicted distribution of Ebola virus disease across Africa	Users can zoom into their country of interest and visualize subnational areas (down to a 5 x 5 km pixel level) that are most environmentally suitable.	Countries can allocate resources, raise awareness, and train health workers in areas of high risk.	http://vizhub.healthdata.org/ebola/; choose country of interest from dropdown menu
Bat distribution layers	Users can choose between 3 RNA-positive bat species, or a combined distribution layer, to understand the environmental suitability for these potential reservoir species (*5*).	Animal health experts can prioritize sampling areas within their countries for EVD surveillance.	http://vizhub.healthdata.org/ebola/; choose bat distribution of interest from indicator dropdown menu or choose country of interest from country dropdown menu, if preferred
Stage 1: spillover risk	Users can explore the relative risk of Ebola virus being transmitted from an animal source to a human, at the district (admin 2) level.	Countries can implement behavior change curriculum in areas at high risk, to inform on the potential risk from wildlife interactions and explain the symptoms of Ebola.	Visualization tool in progress; contact authors for access
Stage 2: local outbreak risk	Users can explore the risk for an index case expanding to a local outbreak, at the district (admin 2) level.	Countries can allocate resources to areas of high risk, strengthening local capacity to ensure index cases are managed and contained effectively.	Visualization tool in progress; contact authors for access
Stage 3: widespread outbreak risk	Users can explore the risk for a local outbreak spreading to neighboring regions, at the district (admin 2) level.	Countries can ensure emergency response operations are in place for a possible outbreak, identifying where quarantine restrictions may be required, as well as where expanded surveillance activities should be undertaken.	Visualization tool in progress; contact authors for access.

*EVD, Ebola virus disease.

The Institute for Health Metrics and Evaluation (IHME) in collaboration with INDEPTH network hosted an Ebola risk mapping and preparedness planning workshop for the countries identified as at risk for a possible outbreak. The workshop was held on February 25 and 26, 2016, in Accra, Ghana, and was attended by participants from 19 at-risk countries across Africa (Table 2).

**Table 2:** Participants from 19 African countries and a variety of animal and human health backgrounds attended the Ebola workshop in Accra, Ghana, on February 25 and 26, 2016

Countries represented	No. participants	Participant sector (field of expertise)
Burundi	2	Government (animal health, public health emergency operations)
Cameroon	2	Government (animal health); nonprofit (animal health)
Central African Republic	2	Government (animal health); nonprofit (public health, infectious disease research)
Republic of Congo	2	Government (animal health); university hospital (human health)
Côte d’Ivoire	3	Government (animal health); nonprofit (public health disease surveillance)
Democratic Republic of the Congo	3	Nonprofit (biomedical research); university (public health, infectious disease research)
Ethiopia	3	Government (public health); university (public health)
Gabon	3	Government (animal health, public health)
Ghana	8	Government (animal health, public health, disease surveillance); university (veterinary medicine, zoonotic diseases); nonprofit (public health research)
Guinea	1	Government (public health, biostatistics)
Liberia	3	Government (public health, public health emergency operations); hospital (human health)
Malawi	3	Government (public health, animal health)
Nigeria	3	Government (public health); university (public health, animal health)
Sierra Leone	5	Government (public health emergency operations, animal health); non-profit (community health); university (environmental health)
South Sudan	1	Government (animal health)
Sudan	1	Government (animal health)
Tanzania	1	Nonprofit (public health research)
Togo	2	Government (animal health)
Uganda	3	Government (public health, infectious disease research, animal health)

## Key Objectives of the Workshop

The key objective of this workshop was to provide in-country teams with an evidence-based tool to understand the risk for an Ebola index case and the capacity to respond and manage any subsequent outbreak. A second objective was to encourage discourse and mutual collaboration across Africa and between animal and human health decision-makers. Participants from a range of backgrounds with expertise on different aspects of Ebola epidemiology were invited to provide a well-rounded discussion of preparedness planning efforts. Discussion topics spanned from animal surveillance efforts to health behavior and education to health system strengthening.

## Agenda

The first day of the workshop focused on estimating the zoonotic niche of Ebola. Richard Suu-Ire from the Forestry Commission in Ghana spoke about his experience sampling bat species in Ghana. Although Ghana has not had a case of EVD in humans, Suu-Ire and his colleagues have identified Ebola virus–seropositive bats in Ghana (*9*), suggesting the potential for spillover infection. Sampling bats is essential for EVD preparedness planning, so an interactive map that could assist in pinpointing sampling sites was presented to workshop attendees (http://vizhub.healthdata.org/ebola/). Using this tool, as well as Google Earth layers, participants determined national sampling sites specific to their region and formed groups with other regional members to discuss their rationale for site selection.

The second day of the workshop focused on the social influences that may affect the scale of any subsequent outbreak. The online map included metrics of travel time, population vulnerability, and infrastructural coping capacity (expanding on a preexisting risk quantification approach) (*10*), enabling identification of areas at greater risk for an index case, local outbreak, and widespread outbreak. Participants explored these layers to understand where best to focus public health efforts. Afterwards, teams from Sierra Leone, Liberia, Uganda, and the Democratic Republic of the Congo reported how their countries have responded to past outbreaks, lessons learned, and the current systems in place to control future outbreaks. Finally, participants returned to their regional groups to discuss preparedness planning measures.

## Reception

Persons from a wide range of occupations from countries with varying experiences with Ebola outbreaks were present, which created a culture of knowledge sharing. The collaborative nature of the workshop led to an appreciation for involvement at both the animal and human level and across national borders. To have a sustainable impact on prevention and control of EVD and other zoonotic diseases, countries must integrate into their healthcare systems a one health approach: an approach involving strong interdisciplinary collaboration and an appreciation that animal and human health are interconnected (*11*). The online visualization tool permitted the participants to observe the areas environmentally suitable for bat populations and compare them with the areas where human populations are most vulnerable.

Although the map cannot pinpoint where the next EVD outbreak will start, participants communicated that the online map was especially helpful in understanding the greatest at-risk areas within their own regions. The online map can be used to translate data into useful information for policy makers, who can now prioritize resource allocation in preparation for any potential outbreak. In the tool, susceptible areas can be visualized at a subnational level within a country. The simplicity and interactivity of this tool was favored by participants, who expressed it would be easy to share with a wide audience. A blog post on the IHME website describes in further detail the participant’s feedback on the online tool and workshop (http://www.healthdata.org/acting-data/mapping-ebola-prepare-future-outbreaks).

## Next Steps

The expectation is that participants will return to their countries and use these tools to open up a narrative about Ebola preparedness, leveraging what they learned from both the maps and those countries that have experienced outbreaks. The workshop’s focus on networking fostered a culture of collaboration between animal and human health experts, as well as across countries.

This work opens up an opportunity for broader epidemic preparedness protocols and collaborations to be developed. The West African outbreak showed that there is a potential for these rare and often small-scale outbreaks to spread from local to national and international levels. This workshop laid the foundation for an open dialogue on the best use of geospatial techniques for prevention of pandemics and showed that interactive maps can be useful to decision makers. IHME currently has a publicly available version of the Ebola map (http://vizhub.healthdata.org/ebola/) and plans to expand our work to other zoonoses with pandemic potential (*12*–*15*).
